# Technical advances in flow cytometry-based diagnosis and monitoring of paroxysmal nocturnal hemoglobinuria

**DOI:** 10.1590/S1679-45082016AO3641

**Published:** 2016

**Authors:** Rodolfo Patussi Correia, Laiz Cameirão Bento, Ana Carolina Apelle Bortolucci, Anderson Marega Alexandre, Andressa da Costa Vaz, Daniela Schimidell, Eduardo de Carvalho Pedro, Fabricio Simões Perin, Sonia Tsukasa Nozawa, Cláudio Ernesto Albers Mendes, Rodrigo de Souza Barroso, Nydia Strachman Bacal

**Affiliations:** 1 Hospital Israelita Albert Einstein, São Paulo, SP, Brazil.; 2 Centro de Hematologia de São Paulo, São Paulo, SP, Brazil.

**Keywords:** Hemoglobinuria, paroxysmal/diagnosis, Flow cytometry/methods, Technological development

## Abstract

**Objective:**

To discuss the implementation of technical advances in laboratory diagnosis and monitoring of paroxysmal nocturnal hemoglobinuria for validation of high-sensitivity flow cytometry protocols.

**Methods:**

A retrospective study based on analysis of laboratory data from 745 patient samples submitted to flow cytometry for diagnosis and/or monitoring of paroxysmal nocturnal hemoglobinuria.

**Results:**

Implementation of technical advances reduced test costs and improved flow cytometry resolution for paroxysmal nocturnal hemoglobinuria clone detection.

**Conclusion:**

High-sensitivity flow cytometry allowed more sensitive determination of paroxysmal nocturnal hemoglobinuria clone type and size, particularly in samples with small clones.

## INTRODUCTION

Paroxysmal nocturnal hemoglobinuria (PNH) is a rare clonal hematopoietic stem cell disease affecting all blood cell lineages, with estimated incidence of 1.3 new case per million population/year. The disease is caused by an acquired somatic mutation in the phosphatidylinositol glycan class A (PIGA) gene encoding an enzyme critical to glycophosphatidylinositol (GPI) anchor biosynthesis. Failure to produce a functional enzyme results in total or partial lack of GPI-anchored proteins on cell surfaces.^(1–4)^

Glycophosphatidylinositol-linked proteins decay accelerating factor (DAF; CD55) and membrane inhibitor of reactive lysis (MIRL; CD59) are responsible for clinical manifestations and laboratory changes associated with PNH.^(5,6)^ These proteins protect normal red blood cells from complement-mediated lysis via prevention of C3 convertase activation and inhibition of membrane attack complex formation.^(3)^ Blood cells with decreased CD55 and CD59 expression are therefore sensitive complement-mediated lysis, which ultimately results in clinical manifestation of the disease.

The classic triad of PNH clinical manifestations includes intravascular hemolysis, thrombosis at uncommon anatomical sites and bone marrow failure. The severity of hemolysis reflects PNH clone size and patients with severe hemolysis often require blood transfusions. Hemoglobinuria and decreased white blood cell counts contribute to increased disease severity due to chronic urinary iron loss and greater susceptibility to infection respectively. Thrombotic events are thought to be a poor prognostic indicator and are the major cause of death in PNH patients.^(7)^

Effective management of PNH is based on accurate clinical and laboratory diagnosis.^(7)^ Flow cytometry (FCM) is the gold standard for PNH diagnosis and monitoring, even in patients with small PNH clones. Along with high sensitivity and specificity, the technique is quick to perform and provides qualitative and quantitative analysis of GPI-anchored proteins.^(8)^ However, standardization of FCM technical procedures is critical for accurate and sensitive test results. Validated guidelines and technical procedures for FCM-based diagnosis and monitoring of PNH have been published.^(3,9)^

Up to 2004, PNH investigation in our FCM laboratory was based on CD14, CD55 and CD59 antigen expression using a two-color FCM assay. In December 2012, improvements in analytical phases and final report, introduction of novel phenotypic markers and purchase of new flow cytometers led to PNH detection technique optimization. This paper reports on our 10-year experience with PNH clone detection and recent technical advances that resulted in higher assay resolution and more accurate determination of red blood cell and white blood cell clone type and size, particularly in samples with small clones.

## OBJECTIVE

To discuss technical improvements implemented for validation of high-sensitivity flow cytometry as a paroxysmal nocturnal hemoglobinuria diagnostic and monitoring tool. Comparative analyses of different protocols and related costs are also provided.

## METHODS

### Data collection

Retrospective study of FCM data from 745 patient samples analyzed from January 2004 to June 2014. The data set included 426 female patients aged 1 to 97 years (median age, 43 years). Flow cytometry analysis was aimed at PNH diagnosis or monitoring in all cases.

### Monoclonal antibodies for flow cytometry-based paroxysmal nocturnal hemoglobinuria detection

For many years, PNH detection in our FCM laboratory was based on two-color FCM assays using specific combinations of antibody-fluorochrome conjugates (Chart 1). Keeping pace with technical development, procedures were upgraded, with particular emphasis on the use of novel markers and new equipment (Chart 2).

**Chart 1:** Markers used for flow cytometry-based (Epics XL-MCL and Cytomics FC500; Beckman Coulter) paroxysmal nocturnal hemoglobinuria clone detection prior implementation of technical advances

Target cells	Antibody and fluorochrome	Clone	Manufacturer
Red blood cells (two-color assay)	CD59 FITC	P282E	Beckman Coulter
	CD235a-PE	11E4B-7-6	Beckman Coulter
	CD55 PE	JS11KSC2.3	Beckman Coulter
Neutrophils (two-color assay)	CD59 FITC	P282E	Beckman Coulter
	CD55 PE	JS11KSC2.3	Beckman Coulter
	CD13 PE	366	Coulter Clone
	CD15 FITC	80H5	Beckman Coulter
Monocytes (two-color assay)	CD64 FITC	22	Beckman Coulter
	CD14 PE	MϕP9	Becton Dickinson

FITC: fluorescein isothiocyanate; PE: phycoerythrin.

**Chart 2:** Markers used for flow cytometry-based (Cytomics FC500 and Navios flow cytometer; Beckman Coulter) paroxysmal nocturnal hemoglobinuria clone detection following implementation of technical advances

Target cells	Antibody and fluorochrome	Clone	Manufacturer
Red blood cells (2-color assay)	CD235a-FITC	11E4B-7-6	Beckman Coulter
	CD59-PE	MEM-43	Invitrogen
Neutrophils (4-color assay)	FLAER Alexa Fluor 488	NA	Cedarlane
	CD24 PE	ALB9	Beckman Coulter
	CD45 ECD	J33	Beckman Coulter
	CD15 PE-Cy5	80H5	Beckman Coulter
Monocytes (4-color assay)	FLAER Alexa Fluor 488	NA	Cedarlane
	CD14 PE	RMO52	Beckman Coulter
	CD45 ECD	J33	Beckman Coulter
	CD64 PE-Cy5	22	Beckman Coulter

FITC: fluorescein isothiocyanate; PE: phycoerythrin; FLAER: fluorescein-labeled proaerolysin; PE-Cy5: phycoerythrin-cyanine 5.

All antibody-fluorochrome conjugates were titrated prior to use for determination of optimal antigen-antibody binding saturation concentrations.^(10)^ Compliance with product specifications (*e.g.*, vendors, clones, dilution and titration) should be emphasized.^(3)^

### Flow cytometer set-up

Flow cytometer settings were adjusted according to our standard operating procedures. In red blood cell assays, voltage settings were established using unstained normal peripheral blood for better definition of forward and side scatter position (logarithmic scale) and negative position/voltage in FL1 and FL2 channels. Samples were individually stained with CD235a-FITC (FL1 *versus* FL2) and CD59-PE (FL2 *versus* FL1) for two-color compensation. Combined staining with CD235a-FITC and CD59-PE was used for setting up verification and optimization.

In white blood cell assays, voltage settings for FSC/SSC (linear scale) and negative position in photomultiplier channels were established using unstained normal peripheral blood. Automatic compensation was achieved via individual staining with previously titrated fluorescein-labeled proaerolysin (FLAER) Alexa Fluor 488, CD45PE, CD45ECD, CD45PE-Cy5, and CD45PE-Cy7. Fine tuning was obtained using the combination described for neutrophils and monocytes (Chart 2).

Associations between protocols and diagnostic sensitivity were investigated using the χ^2^. Comparative analysis of protocols was based on the correlation and linear regression tests. The level of significance was set at 5%.

### Technical procedures

**Figure 1:**
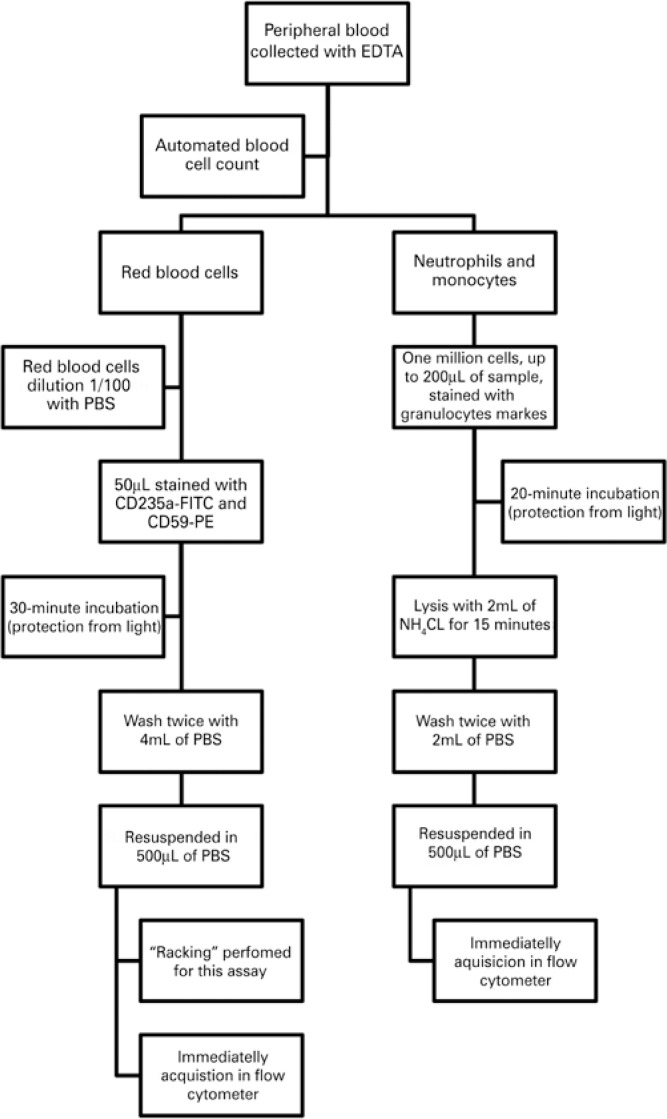
Standardized technical procedures for flow cytometry-based paroxysmal nocturnal hemoglobinuria clone detection EDTA: ethylene diamine tetraacetic acid; PBS: phosphate buffered saline; FITC: fluorescein isothiocyanate; PE: phycoerythrin; NH_4_CL: ammonium chloride.

Technical procedures used for FCM-based PNH clone detection following technical upgrade are summarized in figure 1.

## RESULTS

### Impact of technical advances on flow cytometry assay resolution for paroxysmal nocturnal hemoglobinuria clone detection

Flow cytometry data resolution was crucial to distinguish between normal and PNH populations in mixtures of particles with different signal intensity. Prior to technical upgrade, CD55/CD59 (red blood cell and neutrophils) and CD14 (monocytes) were successfully used to detect PNH clones in our laboratory; however, resolution was poor. Therefore, a normal peripheral blood sample was always stained to serve as reference for identification of PNH positive and negative populations.

**Figure 2:**
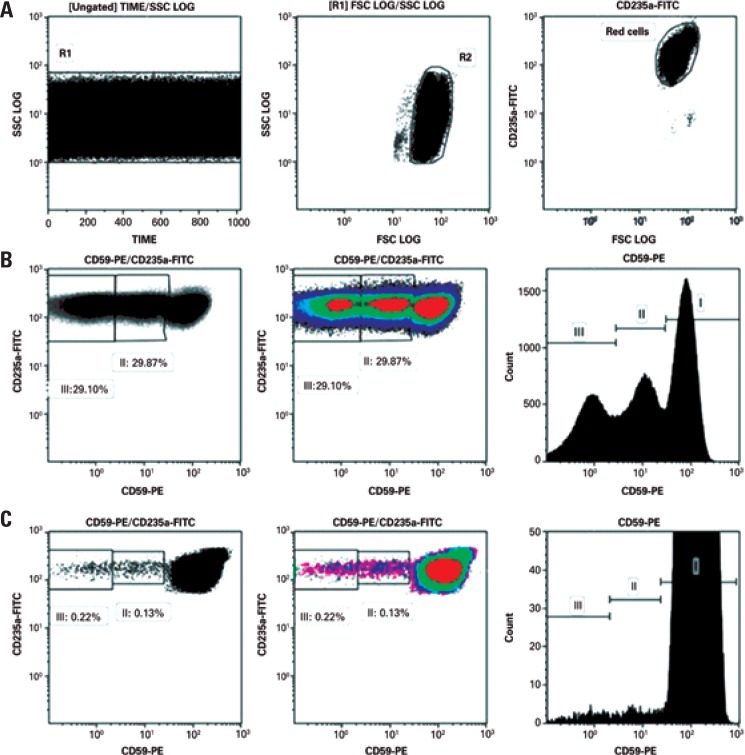
The resolution of flow cytometry data of paroxysmal nocturnal hemoglobinuria investigation in red blood cells lineage after technical improvements. Gating strategies and flow cytometry analysis were based on recommendations given by Sutherland et al.^(11)^ (A) Sequential gating for sensitive detection of CD235a positive red blood cells. This gating strategy is important to eliminate fluidics (SSC log *versus* time) and red blood cell aggregation issues. (B and C) Different paroxysmal nocturnal hemoglobinuria clones based on CD59 expression. Data sets comprising dot plots, density plots and histograms are useful for more sensitive detection and characterization of paroxysmal nocturnal hemoglobinuria clones FSC: forward scatter; SSC: side scatter; FITC: fluorescein isothiocyanate; PE: phycoerythrin.

**Figure 3:**
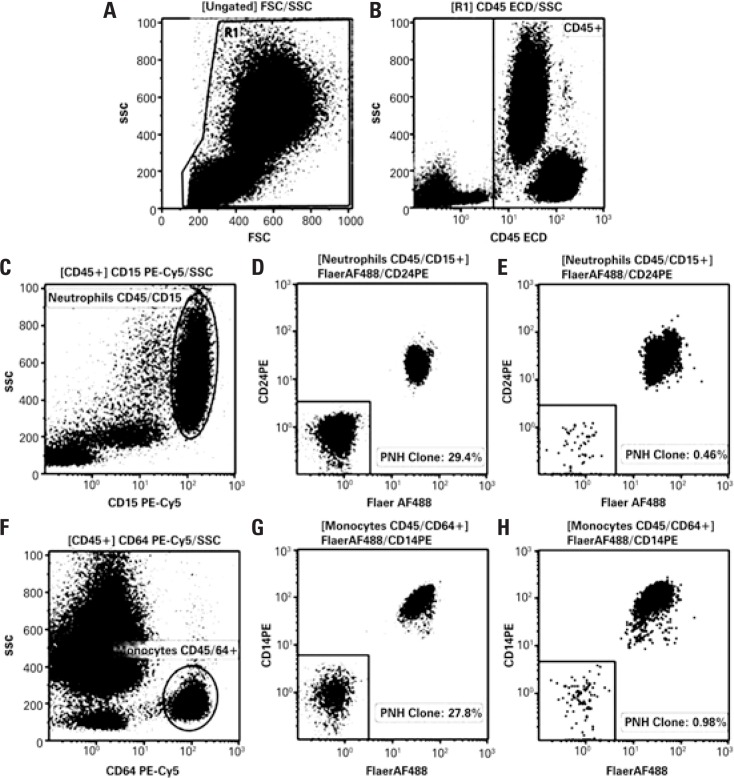
Resolution of flow cytometry data of paroxysmal nocturnal hemoglobinuria investigation in white blood cells lineage after technical improvements. (A, B, C and F) Gating strategies for neutrophil and monocyte identification. (D, E, G and H). Paroxysmal nocturnal hemoglobinuria clone size in CD45/CD15+ neutrophils (D and E) and CD45/CD64+ monocytes (G and H) based on fluorescein-labeled proaerolysin and CD24 and fluorescein-labeled proaerolysin and CD14 expression, respectively FSC: forward scatter; SSC: side scatter; FITC: fluorescein isothiocyanate; PE: phycoerythrin; PNH: paroxysmal nocturnal hemoglobinuria.

Introduction of novel markers (Chart 2) and refinement of technical procedures (Figure 1) enabled high-resolution identification of PNH clones with 0.01% sensitivity and characterization of different clones based on partial or total absence of GPI-linked proteins (Figures 2 and 3).

### Performance of paroxysmal nocturnal hemoglobinuria detection protocols before and after technical upgrade

**Figure 4:**
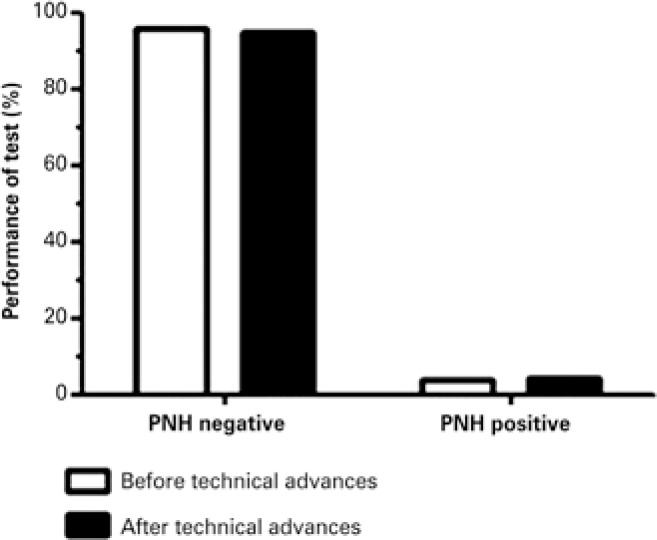
Performance of paroxysmal nocturnal hemoglobinuria detection protocols employed before and after implementation of technical advances. The frequency of negative and positive samples did not differ significantly between protocols (p=0.714) PNH: paroxysmal nocturnal hemoglobinuria.

The number of positive cases before and after implementation of technical advances was evaluated to compare the performance of PNH detection protocols. Only negative and positive results of samples analyzed for diagnostic purposes (*i.e.*, not for monitoring) were considered; 573 out of 745 (76.9%) samples analyzed before technical updates and 172 samples analyzed after technical updates were selected. Paroxysmal noctunal hemoglobunuria clones were detected in 4% (23 out of 573) and 4.7% (8 out of 172) samples analyzed before and after technical improvements respectively. However, differences were not statistically significant (p=0.714) (Figure 4).

### Alternative use of CD157 for paroxysmal nocturnal hemoglobinuria detection

Different GPI-linked proteins can be used for FCM-based PNH investigation. In recent studies using stabilized PNH blood samples, some of these proteins outperformed CD14, CD16, CD24 and FLAER in detecting PNH white blood cell.^(12)^ According to recent publications, CD157 is a potentially useful marker for PNH detection, with high sensitivity and specificity, which could also be used to evaluate GPI-linked protein expression in neutrophils and monocytes in the same reaction.^(13,14)^

For procedure standardization purposes, the use of a single sample tube containing five conjugates for simultaneous analysis of neutrophils and monocytes^(14)^ was validated in our laboratory. Markers are described in chart 3. Cytomics FC500 and Navios flow cytometer (Beckman Coulter) were employed.

**Chart 3:** Single 5-color tube assay for paroxysmal nocturnal hemoglobinuria investigation in white blood cells

Target cells	Antibody and fluorochrome	Clone	Manufacturer
Neutrophils and monocytes (five-color assay)	FLAER Alexa Fluor 488	NA	Cedarlane
	CD157 PE	SY11B5	Exbio
	CD64 ECD	22	Beckman Coulter
	CD15 PE-Cy5	80H5	Beckman Coulter
	CD45 PE-Cy7	J33	Beckman Coulter

FLAER: fluorescein-labeled proaerolysin; NA: not applicable; PE: phycoerythrin; PE-Cy5: phycoerythrin-cyanine 5; PE-Cy7: phycoerythrin-cyanine 7.

**Figure 5:**
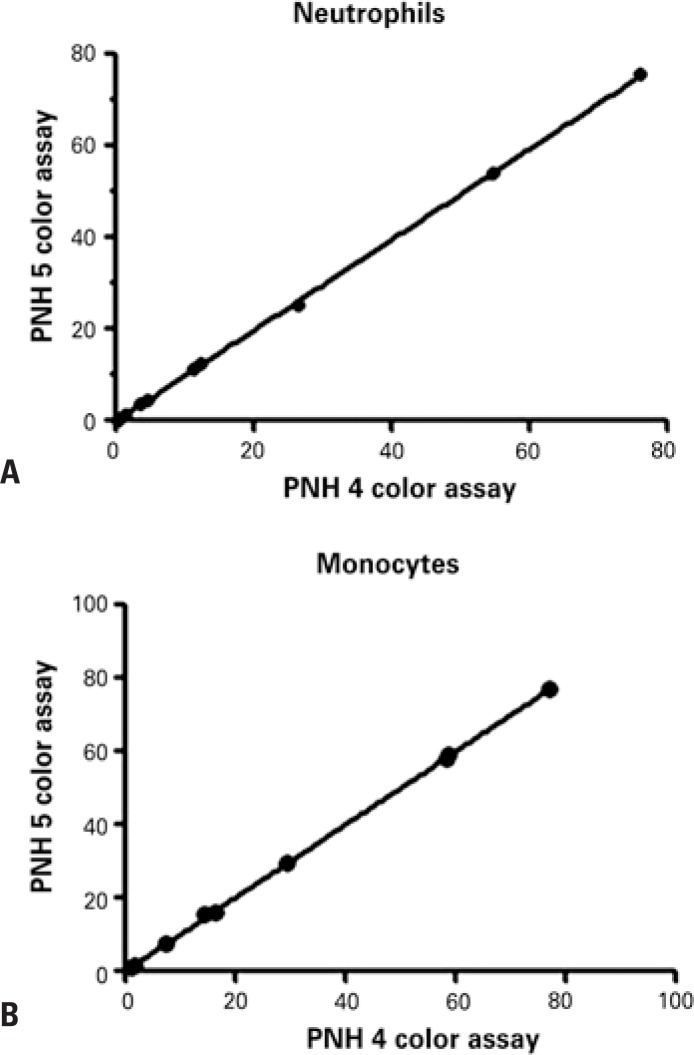
Comparison between paroxysmal nocturnal hemoglobinuria clone size detected by two tubes four-color assay and a single tube five-color assay. (A and B) represent, respectively, the correlation of neutrophils CD15+ and monocytes CD64+(B) with different paroxysmal nocturnal hemoglobinuria clone size. The statistically significance was evaluated using the correlation test and linear regression (R2=0.9998; p<0.0001) PNH: paroxysmal nocturnal hemoglobinuria.

**Figure 6:**
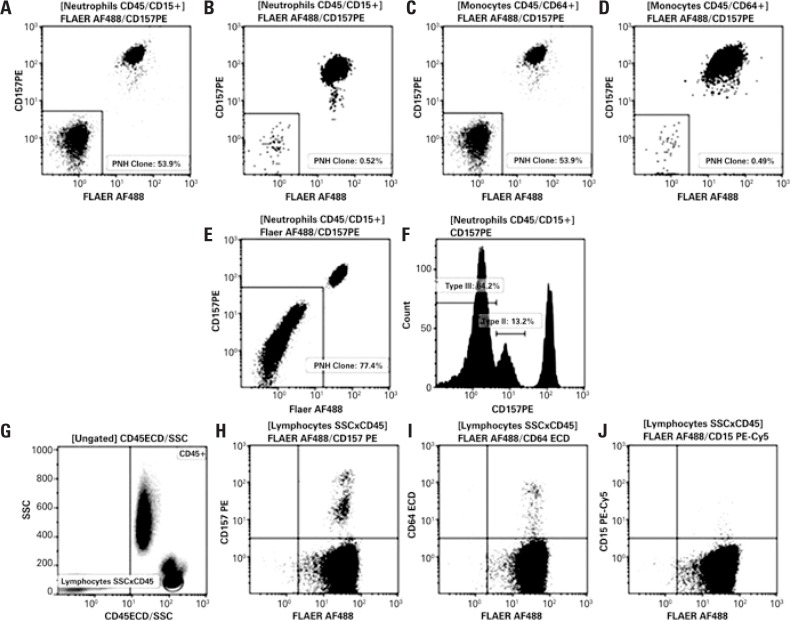
White blood cell analysis using the single tube five-color assay described in chart 3. Gating strategies employed for neutrophil and monocyte identification are described in figure 3. (A, B, C and D) Paroxysmal nocturnal hemoglobinuria clone size in CD45/CD15+ neutrophils (A and B) and CD45/CD64+ monocytes (C and D) based on fluorescein-labeled proaerolysin and CD157 expression. (E and F) Sample containing types II and III paroxysmal nocturnal hemoglobinuria clones detected in white blood cell lineages using fluorescein-labeled proaerolysin and CD157-based assays. (G, H I and J) Lymphocytes (SSCxCD45) used as internal control for evaluation of technical procedures, reagent performance and flow cytometer settings PE: phycoerythrin; PNH: paroxysmal nocturnal hemoglobinuria.

Twenty samples were submitted to flow cytometric analysis using the current two tubes four-color assay and the single tube five-color assay for performance comparison. Different clone sizes were detected in neutrophils (0.2% to 76.0%) and monocytes (0.9% to 77.0%) in ten samples. Statistically similar PNH clone sizes were detected with both methods (Figures 5 and 6). The remaining ten samples tested negative in both assays.

Samples obtained from an external quality program (United Kingdom National External Quality Assessment Service; UK NEQAS) were also evaluated for test validation. As an example, FLAER and CD157-based assays revealed 0.12% cell in CD15^+^ neutrophils in one of these samples. Results were consistent with the 0.08% to 0.13% range proposed in PNH UK NEQAS trials.

### Impact of technical advances on paroxysmal nocturnal hemoglobinuria test costs

To assess the impact of technical upgrade on test costs, the three above described scenarios were analyzed using activity-based costing (ABC).^(15)^ This method eliminates profitability variations and establishes pricing policies based on costs and processes, including variable cost (supplies, with fractioning and waste estimation), fixed cost (medical and technical personnel, infrastructure, depreciation) and administrative cost analysis.

Important variables, such as laser time, FCM data acquisition and analysis, reagent and supply costs, and technical and medical personnel workload and service costs were taken into account in comparative analysis of technical procedures.

**Figure 7:**
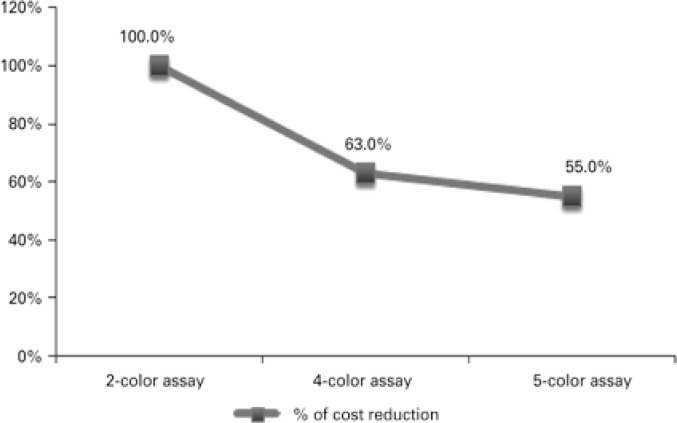
Activity-based costing. Paroxysmal nocturnal hemoglobinuria test cost reduction was associated with technical procedures. The two-color assay was taken as the starting point for comparative analysis of technical procedures in this study (two tubes four-color and single tube five-color assays)

Results revealed significant cost reduction. Two-color assay costs were 37.0% and 45.0% higher compared to four-color assay and single tube five-color assay costs respectively. Four-color assay costs were 12.7% higher than single tube five-color assay costs (Figure 7).

## DISCUSSION

This retrospective study reports on our 10-year experience in FCM-based PNH clone detection and discusses recent technical advances, which resulted in higher resolution and sensitivity in small PNH clone detection. To this end, recommendations given in international guidelines were complied with and technical procedures upgraded accordingly.^(3,8,9,14,16–18)^

In white blood cell assays, FLAER brought the major breakthrough in diagnosis and even more so in monitoring of PNH clones, given the higher accuracy and sensitivity of this marker. FLAER is thought to be able to detect small clones to a level of approximately 0.5%,^(17)^ particularly in cases of severe neutropenia, aplastic anemia (AA) and myelodysplastic syndromes (MDS).^(19–21)^ Replacement of CD55/CD59 with FLAER/CD14 (monocytes) or FLAER/CD24 (neutrophils) in our procedures improved FCM data resolution (Figures 3E and 3H), particularly of small sized clones.

A well-established high-sensitivity single tube five-color assay for PNH leukocyte detection^(14)^ was recently validated in our FCM laboratory (Figure 6). Comparative analysis of 20 samples using this and the two tubes four-colour assay revealed high levels of correlation (Figure 5). Similar PNH clone sizes were detected in 10 out of 20 samples analyzed using both methods. No clones were detected in the remaining ten samples with either method.

Interestingly, CD157 was able to detect type II PNH leukocytes (Figures 6E to 6F). Similar findings have been reported elsewhere.^(3,14)^ However, their clinical significance remains to be determined.^(3,9,14)^ Type II PNH leukocyte detection is not limited to the use of CD157; markers such as CD55, CD59, CD24 (neutrophils) and CD14 (monocytes) can also be employed.

The major technical challenge in red blood cell analysis is the combination of different antibody conjugates without inducing significant erythrocyte aggregation, which could interfere with detection of types II and III PNH clones. Although CD55 and CD59 are traditionally used, CD235a-FITC and CD59-PE conjugate antibodies are the best established markers.^(9)^ The use of this combination improved FCM data resolution in our red blood cell analysis (Figure 2). Prior to introduction of technical updates such as proper titration and dilution of red blood cell markers and racking of samples before data acquisition, discrimination between types II and III PNH clones was not possible, not even in small clones.

Type II and III PNH red blood cell have different sensitivity to the lytic action of complement^(22)^ and their correct identification is associated with disease clinical manifestations and symptoms. Clinical manifestations associated with intravascular hemolysis have been reported in patients with more than 20% of PNH type III cells.^(3)^

Clone size monitoring is important for patient management. Monitoring of cases with evidences of small clone size requires high-resolution and high-sensitivity FCM assays. The technique described in this article is able to detect small PNH clones (Figures 3E to 3H and Figures 6B to 6D), in that one million events are acquired with 0.01% sensitivity.^(16)^ However, large clones can be adequately detected and monitored using assays with good precision and low-sensitivity.

Detection of small and subclinical PNH clones in patients with AA and MDS has been reported.^(7,21)^ Clone size monitoring is important because some patients (AA patients in particular) may respond to immunosuppressive therapy; also, PNH clone expansion may lead to PNH-related clinical manifestations.^(19,20)^

Along with the introduction of technical advances, FCM protocol refinement translated into significant reduction in PNH test costs (activity-based costing; Figure 7). Cost reduction was associated with shorter sample preparation time, faster FCM data acquisition and analysis, reduced cost of reagents and supplies and reduced technical and medical staff workload and service costs. Cost reduction could not be translated into currency out of confidentiality concerns. However, supposing the starting point for comparative analysis (two-color assay; Figure 7) corresponds to 100%, a given value in Brazilian currency (*e.g.*, R$ 1,00), could be attributed to it for calculation of cost reduction in equivalent monetary value. Also, normal peripheral blood was replaced with negative and positive PNH populations as internal control for evaluation of technical procedures, antibody/reaction performance and FCM settings (Figures 6G to 6J).

Our laboratory joined the UK NEQAS and College of American Pathology external quality programs in November 2004 and April 2006, respectively. In April 2013 we also joined the UK NEQAS PNH High-Resolution program. The role of these programs in PNH screening test quality, performance and sensitivity assessment should be emphasized. Corrective action and continuous improvement based on external program outcomes translated into efficient refinement of our FCM-based PNH detection methods.

## CONCLUSION

Flow cytometry-based methods used for detection of paroxysmal nocturnal hemoglobinuria clones in our laboratory are upgraded according to recommendations given in literature. Guidelines for technical procedures, data analysis and interpretation, final report preparation and reliability assessment are complied with. Implementation of novel methodological strategies for diagnosis and/or monitoring of paroxysmal nocturnal hemoglobinuria resulted in improved resolution and detection of red blood cell and white blood cell clones, with more accurate determination of clone type and size, particularly in samples containing small clones (high-sensitivity flow cytometry). Significantly reduction in assay costs was also achieved.
